# Molecular Aberrations Stratify Grade 2 Astrocytomas Into Several Rare Entities: Prognostic and Therapeutic Implications

**DOI:** 10.3389/fonc.2022.866623

**Published:** 2022-06-10

**Authors:** Valeria Internò, Giacomo Triggiano, Pierluigi De Santis, Luigia Stefania Stucci, Marco Tucci, Camillo Porta

**Affiliations:** ^1^Department of Interdisciplinary Medicine, University of Bari ‘Aldo Moro’, Bari, Italy; ^2^Division of Medical Oncology, Policlinico Hospital of Bari, Bari, Italy

**Keywords:** diffuse low-grade gliomas, prognostic molecular stratification, *IDH*-wt grade 2 astrocytoma, *pTERT* mutation, *EGFR* amplification

## Abstract

The identification of specific molecular aberrations guides the prognostic stratification and management of grade 2 astrocytomas. Mutations in isocitrate dehydrogenase (*IDH*) 1 and 2, found in the majority of adult diffuse low-grade glioma (DLGG), seem to relate to a favorable prognosis compared to *IDH* wild-type (*IDH*-wt) counterparts. Moreover, the *IDH*-wt group can develop additional molecular alterations worsening the prognosis, such as epidermal growth factor receptor amplification (*EGFR-*amp) and mutation of the promoter of telomerase reverse transcriptase (*pTERT-mut*). This review analyzes the prognostic impact and therapeutic implications of genetic alterations in adult LGG.

## 1 Introduction

Grade 2 astrocytomas are rare tumors occurring in almost 1 out of 200,000 people per year with a peak incidence of between 30 and 35 years (1–4). Since 2016, neuro-pathologists introduced genetic parameters to differentiate them from oligodendrogliomas (5–8). Due to their slow growth, they determine cortical adaptor mechanisms leading to a functional and morphologic brain reorganization; as a consequence, tumor onset is usually characterized by seizures in the absence of other neurological deficits (9–11). Grade 2 astrocytomas are prognostically differentiated depending on the presence or absence of isocitrate dehydrogenase (*IDH*) 1 and 2 mutations, which, when occurring, correlate to a favorable outcome. Additionally, the *IDH*-wt patient’s prognosis worsens in presence of *pTERT*-mutations (*pTERT*-mut) and *EGFR* amplification (*EGFR*-amp) (12, 13). The treatment of lower-grade astrocytomas is established according to several stratification features (anaplastic gliomas, patients aged over 40 years, and subtotal removal). In presence of at least one of those risk factors, the suggested treatment is multimodal, consisting of post-surgical radiation therapy followed by chemotherapy with either temozolomide or a combination of procarbazine, lomustine, and vincristine (PCV). Due to limited available evidence, the predictive role of the molecular landscape of these tumors is still debated and not fully understood. However, both *pTERT*-mut and *EGFR* amplification (*EGFR*-amp) in IDH-wt tumors seem to identify a subgroup of patients not benefiting from adjuvant therapies (13–15).

Presently, there is no certainty about adequate therapeutic strategies for different molecular subtypes of grade 2 astrocytomas. Thus, in this review, we realize a comprehensive overview of the prognostic role of molecular aberrations and the most effective post-surgical strategies.

## 2 The Backbone of Diffuse Low-Grade Glioma Molecular Alterations: *IDH* Mutation

*IDH1/2*-mut gliomas usually harbor genetic and clinical characteristics conferring them to be a better outcome with respect to their *IDH*-wt counterpart. *IDH* mutations, usually localized at the arginine residue (R132 for IDH1, R140, or R172 for IDH2), are somatic heterozygous and missense point mutations producing the oncometabolite d-2-hydroxyglutarate (D-2HG) and promoting the transformation in immortalized human astrocytes (8). Although an early molecular event, *IDH* mutation is not sufficient to generate gliomas; further molecular alterations are required (16–19). Precisely, when *IDH1*-mutated, astrocytomas are often associated with *TP53* mutation, while oligodendrogliomas present loss of 1p/19q and very rarely *TP53* mutation. Therefore, three different molecular pathways can be identified, the first one arising with the mutation of *IDH* followed by *TP53* mutation, which generates grade 2 astrocytomas. The second one involves grade 2 oligodendrogliomas, characterized by *IDH* mutation, followed by the loss of 1p and 19q. The last pathway includes gliomas without mutations in *IDH* gene, but with multiple genetic alterations, such as amplification or mutation of *EGFR* and loss of *PTEN* gene. This last subgroup of tumors becomes early aggressive glioblastoma (GBM) (20, 21).

## 3 Beyond *IDH* Mutation: Molecular-Guided Glioma Classifications

### 3.1 WHO Classification of Tumors of the Central Nervous System, 2016 Classification (WHO 2016CNS)

**Figure 1:**
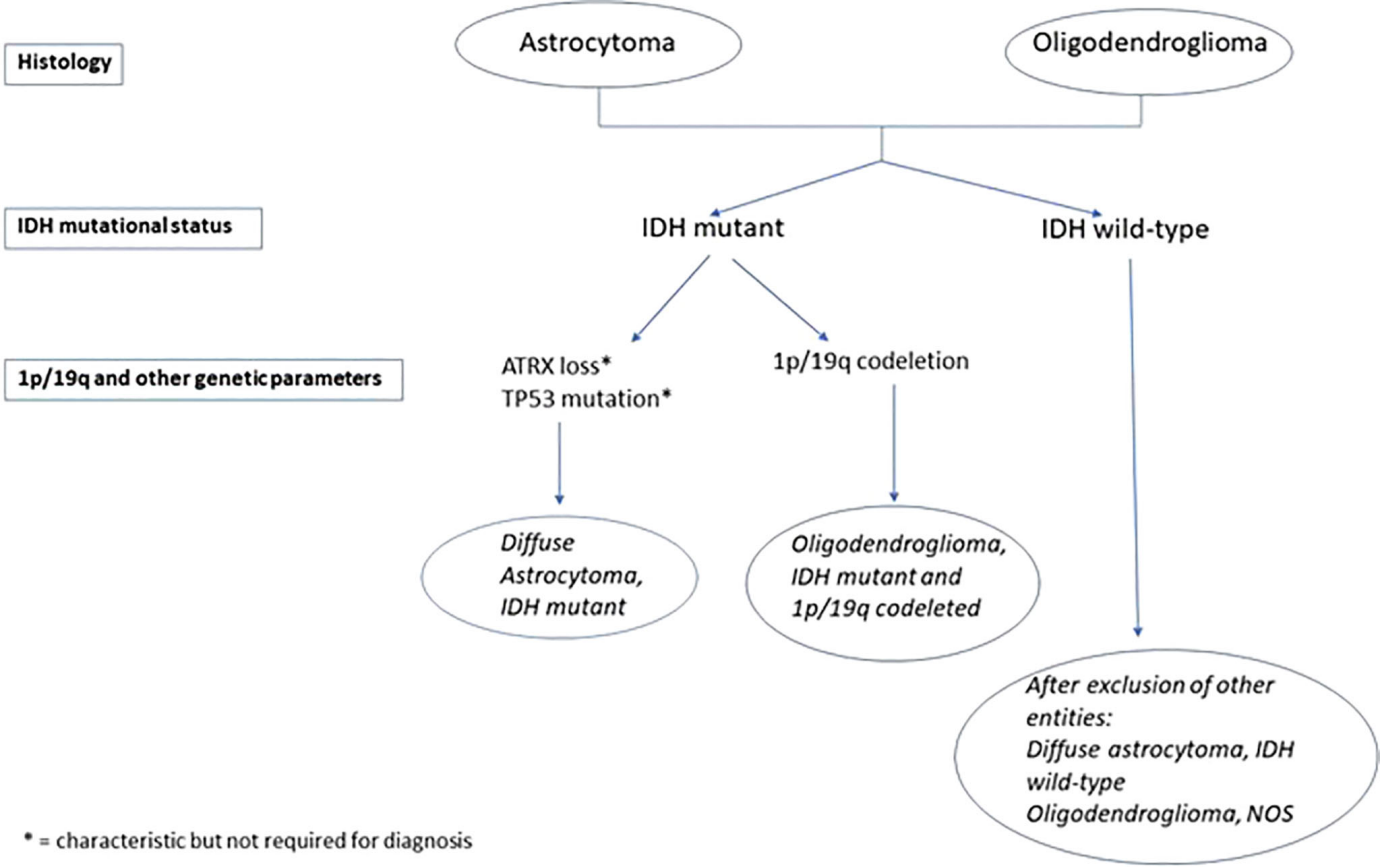
Molecular pathways of IDH-mut lower-grade gliomas development. *IDH1*/2 mutations are early events in glial progenitors after which these cells acquire additional mutations: *ATRX* and *TP53* mutations in astrocytomas, and 1p/19q co-deletion and *pTERT* mutations in oligodendrogliomas.

**Figure 2:**
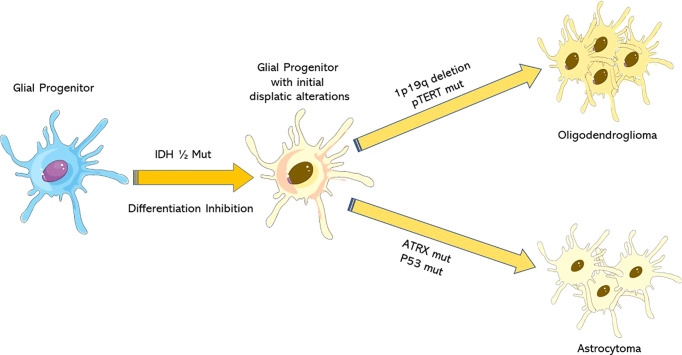
WHOCNS2016 algorithm for diffuse low-grade glioma (DLGG) diagnosis. A scheme of molecular analysis is needed to formulate a precise diagnosis of DLGG, according to WHOCNS2016.

For the first time, WHO2016CNS stratified diffuse low-grade glioma (DLGG) into separate entities according to molecular parameters: *IDH-*mut, *IDH*-wt, and not otherwise specified (NOS) categories, as evidenced in **Figures 1** and **2** (16–24).

### 3.2 cIMPACT-NOW Updates 3 and 5

In 2016, a Consortium to Inform Molecular and Practical Approaches to CNS Tumor Taxonomy (cIMPACT-NOW) was created to update neuro-oncologists on the novel insights of the DLGG molecular landscape. In particular, the third and fifth updates focused on adult *IDH*-wt and *IDH*-mut diffuse astrocytic gliomas (25–28). The first one concerned a specific subset of *IDH*-wt diffuse or anaplastic astrocytomas prognostically similar to grade 4 *IDH*-wt GBM (27, 29–34). Precisely, it was established that at least one of the following alterations is necessary to compare them to a GBM since the lack of *IDH* mutation is not sufficient to confer a worse prognosis: *EGFR*-amp, *pTERT-*mut, the combined chromosome 7 gain, and chromosome 10 loss. These entities rarely occur in grade 2 astrocytoma, belonging mainly to grade 3 (35–40). However, *pTERT-*mut, when not associated with the abovementioned alterations, may relate to *IDH*-wt gliomas with favorable prognosis (pleomorphic xanthoastrocytoma, ganglioglioma, and nearly all oligodendrogliomas) (34, 38, 41). The fifth update focused on *IDH*-mut DLGG prognostic molecular features and identified the following alterations conferring the worse impact: *CDKN2A/B* homozygous deletion, *CDK4* amplification, *RB1* mutation or homozygous deletion, *PIK3CA* or *PIK3R1* mutations, *PDGFRA* amplification, *MYCN* amplification, global DNA methylation levels, genomic instability, and chromosome 14 loss, as listed in **Table 1**.

**Table 1:** Genetic aberrations conferring an aggressive behavior to grade 2 astrocytomas.

Molecular alterations with a poor prognostic role in grade 2 astrocytomas	
IDH-mut	IDH-wt
CDKN2A/B homozygous deletion	EGFR amplification
CDK4 amplification	pTERT mutation
Chromosome 14 loss	Chromosome 7 gain and chromosome 10 loss
G-CIMP-low DNA methylation pattern	
PIK3CA mutation	
MYCN amplification	

Several studies evidenced the role of *CDKN2A/B* homozygous deletion as an independent poor prognostic factor in *IDH*-mut diffuse astrocytomas (42–44). Further analyses showed that *CDK4* amplifications are common among *IDH*-mut astrocytomas with poor prognosis, and their combination with chromosome 14 loss predicted an even shorter overall survival (OS) (40, 45). Moreover, mutations in *PIK3R1* and *PIK3CA* genes, as well as amplification in *MYCN* and genomic instability, are associated with a worse outcome (32). In conclusion, *IDH*-mut lower-grade astrocytomas can be split into 3 different prognostic subgroups. The first one shows the best prognosis (median OS (mOS) greater than 10 years) with no evidence of mitotic activity, histologic anaplasia, microvascular proliferation, necrosis, or *CDKN2A/B* homozygous deletion (46). The second one, with a shorter life expectancy (nearly 8 years), showed the presence of mitosis and anaplasia, but neither microvascular proliferation nor necrosis nor *CDKN2A/B* homozygous deletion (47). The third subgroup corresponds to WHO grade 4 gliomas when at least one of the following features is identified: microvascular proliferation, necrosis, or *CDKN2A/B* homozygous deletion. Moreover, it is currently classified as astrocytoma *IDH*-mut; grade 4 causes its outcome results to be more favorable compared to GBM *IDH*-wt one (32–48).

### 3.3 CNS WHO 2021 Classification, Fifth Edition (WHO CNS5)

WHO CNS Classification’s fifth edition (WHO CNS5), published in 2021, is the most practice-changing one, mainly focusing on the molecular alterations’ role (rather than on the morphological analysis) considered as biomarkers of grading and for further estimating prognosis. According to this classification, GBM diagnosis can be formulated even in presence of a histologically lower-grade glioma, if including one of the following alterations: *CDKN2A/B* homozygous deletion in *IDH*-mut astrocytomas, as well as p*TERT*-mut, *EGFR-*amp, and +7/−10 copy number changes in *IDH*-wt diffuse astrocytomas (49). Therefore, WHO CNS5 identifies only 3 types of gliomas: astrocytoma, *IDH-*mut; oligodendroglioma, *IDH*-mut and 1p/19q-codeleted; and GBM, *IDH*-wt.

In other words, the current classification aims to add value to molecular parameters compared to histological findings in defining the tumoral grade. Due to the new 2021 WHO classification way of diagnosing lower-grade gliomas (different from the 2016 WHO still widely used), an Expert Panel of the American Society of Clinical Oncology (ASCO) and Society for Neuro-Oncology (SNO) published guidelines concerning the therapeutic management of diffuse astrocytic and oligodendroglial tumors in adults in order to implement both of them, till the new classification system will be definitively adopted (50).

## 4 Clinical-Therapeutic Implications of Grade 2 Astrocytomas Molecular Landscapes

### 4.1 *IDH*-mut Grade 2 Astrocytomas

#### 4.1.1 Clinical and Radiological Features

Approximately 80% of newly diagnosed astrocytomas harbor *IDH* mutations. Survival differences between *IDH*-mut and *IDH*-wt LGG (mOS 10 vs. 2.1 years) led to separate them since WHOCNS2016 (8). Clinical and radiological peculiarities help discern between the abovementioned entities; for instance, epilepsy is frequently evidenced in *IDH*-mut DLGG due to the biological activity of D2HG, which is the product of the mutant enzyme. *IDH1*-mut cells expose neurons to D2HG, which structurally resembles glutamate, and disrupt the balance between inhibition and excitation, leading to seizures (51). Radiologically, MR spectroscopy might discriminate between *IDH*-mut and *IDH*-wt astrocytomas by quantifying 2-HG, detected only in *IDH*-mut gliomas (8). Moreover, *IDH*-mut astrocytomas display a characteristic T2 fluid-attenuated inversion recovery (T2-FLAIR) mismatch sign, defined as the presence of complete/near-complete hyperintense signals on a T2-weighted image, and a relatively hypointense signal on FLAIR, except for a hyperintense peripheral rim, with a specificity rate ranging at 100% (52).

#### 4.1.2 Role of Surgery

The need for tumor specimens for molecular analysis has further increased the indications for neurosurgery. Its indications and extent of resection (EOR) depend on tumor-specific factors; observation is usually reserved in the presence of comorbidities contraindicating surgical approach, and biopsy in the case of tumors located in delicate areas or the presence of disseminated, multicentric, and/or bulky gliomas (53, 54). Indeed, resection needs to be maximally radical to reach an oncological efficacy, with EOR positively influencing both progression-free survival (PFS) and OS. EOR can be quantified and identified as biopsy, subtotal resection (STR), gross total resection (GTR), and supratotal resection (SuTR) (55). GTR seems to be more feasible for *IDH*-mut low-grade astrocytomas due to their less infiltrative behavior, conferring longer survival and benefits in terms of seizure control (56).

#### 4.1.3 Adjuvant Therapies

**Figure 3:**
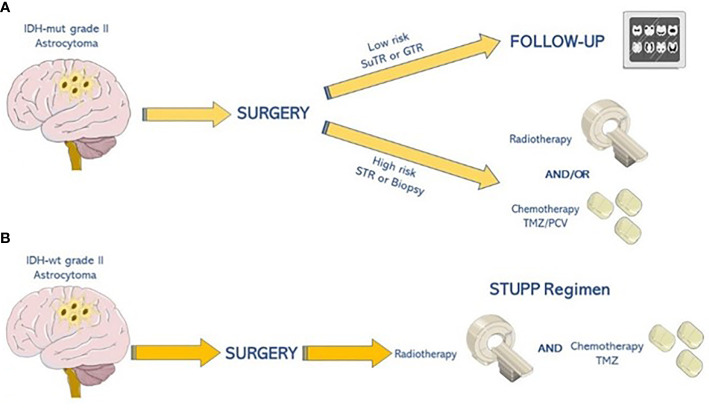
Therapeutic algorithm for grade 2 astrocytoma. **(A)** Patients with IDH-mut grade 2 astrocytomas are divided into two categories (low-risk versus highrisk). Low-risk patients should undergo radiological follow-up, while high-risk ones are eligible for adjuvant therapies, such as radiotherapy (50–54 Gray in 1.8 Gray/fraction) followed by chemotherapy alone with temozolomide (TMZ) or procarbazine, lomustine, and vincristine (PCV). **(B)** Patients with IDH-wt astrocytomas undergo concomitant and adjuvant regimens based on radio-chemotherapy with temozolomide as STUPP regimen.

As shown in **Figure 3**, post-surgical strategies have been established by analyzing the long-term results of several phases III clinical trials, which started back in the 1990s by the European Organisation for Research and Treatment of Cancer/Radiation Therapy Oncology Group (EORTC/RTOG). Patients with *IDH*-mut lower-grade astrocytomas are divided into two categories (low versus high risk) based on the EOR and/or unfavorable prognostic factors (i.e., age older than 40 years, presence of neurological deficits, and uncontrolled seizures). Low-risk patients undergo radiological follow-up without any adjuvant treatment. On the contrary, high-risk patients are directed to adjuvant treatments, such as radiotherapy (RT) alone (50–54 Gray in 1.8 Gray/fraction), chemotherapy alone (with temozolomide or PCV), or a combination of the two approaches (57). In particular, the EORTC 22845 trial compared postoperative RT to RT performed at the time of disease progression in adults with grade 2 gliomas. A significantly better PFS was evidenced in the first group of patients. Conversely, there was no significant difference in terms of OS (58). Moreover, the RTOG 9802 study evaluated RT alone versus RT followed by PCV in high-risk low-grade gliomas. Both PFS and OS were significantly improved in the combination arm, with a gain of 5.5 years on survival (13.3 versus 7.8 years) (59). Finally, the EORTC 22033 trial showed no significant differences in terms of PFS in upfront RT versus dose-dense temozolomide (75 mg/m^2^ daily on a 21/28 days scheme) in high-risk, grade 2 gliomas. Data on OS are not yet available. Further results concerning OS will help clarify the adequate adjuvant therapy for *IDH*-mut grade 2 astrocytomas (60).

### 4.2 *IDH*-wt, Morphologically Grade 2, Astrocytomas

#### 4.2.1 Genomic Landscape and Risk Assessment

*IDH*-wt grade 2 astrocytomas (WHO 2016) usually harbor GBM’s molecular alterations, leading to consider them as an immature GBM still missing microvascular proliferation and necrosis (36, 61–63). However, the outcome of *IDH*-wt astrocytomas is strictly related to several genetic features such as K27M mutation of histone 3 family 3A (*H3F3A*; *H3-K27M*); V600E mutation of B-rapidly accelerated fibrosarcoma (*BRAF*); *pTERT*-mut; *EGFR*-amp; and chromosome 7 gain, and chromosome 10 loss (64–67). Three retrospective studies evaluated *IDH*-wt grade 3 astrocytomas, and only one focused on grade 2 astrocytomas (68, 69). The first one collected 718 LGG including 166 *IDH*-wt gliomas. *EGFR-*amp, *BRAF*, and *H3F3A* mutations were observed in a mutually exclusive pattern in 13.8%, 6.9%, and 9.5% of patients, respectively. *pTERT* mutations were evidenced in 26.8% of specimens. Patients younger than 45 years of age with grade 2 oligodendroglioma showed the most favorable prognosis. On the contrary, older patients with anaplastic gliomas who underwent STR, with *EGFR-*amp and *H3F3A* mutation, experienced shorter survival. Furthermore, gliomas were divided into “molecularly” low- and high-grade, based on the absence or presence of *EGFR*, *H3F3A*, or *pTERT* gene alterations; the first group showed a mOS improvement of about 6 months. Notably, the most favorable outcome was evidenced in molecularly low-grade gliomas with *MYB* amplification (68). The second study, published by Wijnenga and colleagues, confirmed the detection of *pTERT-*mut or chromosome 7 gain and chromosome 10 loss status as poor prognostic factors (39). The third retrospective study explored molecular alterations in 160 *IDH*-wt gliomas divided into 120 anaplastic and 40 grade 2 astrocytomas. The authors identified four molecularly driven subgroups: 78% considered conventional GBM due to the same molecular aberrations, the second group (9% of patients) showed a mutation in *H3F3A* gene, and the third one (8%) shared the methylation profile with GBM-*H3-K27* mutated. Finally, the last group (5%) showed a molecular profile similar to the large cellular GBM (68). The latter study, published in 2019, evaluated 35 patients with *IDH*-wt lower-grade astrocytomas confirming the negative prognostic role of chromosome 7 gain and chromosome 10 loss and *pTERT-*mut. mOS was shorter in patients with at least one of these negative prognostic factors (18.5 vs. 54.5 months) (68). Recently, literature data highlighted that the presence of *pTERT*-mut alone could not be enough to call molecular GBM a grade 2 *IDH*-wt glioma (69).

#### 4.2.2 Role of Surgery

EOR prognostic impact in *IDH*-wt grade 2 astrocytomas is still being debated because randomized controlled trials are still missing. However, a systematic review published in 2020 considered maximal resection with preservation of eloquent brain areas an essential treatment strategy in terms of survival benefits (70). Moreover, Poulen et al. performed a retrospective analysis of 31 patients receiving a high rate of maximal resection (nearly 95%) not followed by any postoperative adjuvant treatment. In their population, 5 patients underwent STR dying rapidly (3.5 years from diagnosis), while all patients undergoing GTR are still alive after a 5-year follow-up. So GTR may improve outcomes even in *IDH*-wt patients. On the contrary, a retrospective single-center study from Patel et al. found no association between EOR and outcome in 25 *IDH*-wt grade 2 astrocytomas out of 172 LGG patients (71).

#### 4.2.3 Adjuvant Therapies

There is still a paucity of evidence about post-surgical strategies in this setting. Treatment should be decided considering patient-specified prognostic factors such as age, Karnofsky Performance Status (KPS), molecular profile, clinical and radiological course, and MGMT promoter methylation status. Usually, due to expected poor prognosis, concomitant RT and temozolomide-based chemotherapy are often carried out even if never formally evaluated within randomized controlled trials. In particular, RT sensitivity has never been established and might be lower due to wild-type *IDH* enzymes’ protective effect through the maintenance of NADPH levels counteracting apoptosis (72). EORTC 22033 and 26033 studies suggest the Stupp regimen as the most adequate treatment, due to the aggressiveness of the majority of *IDH*-wt grade 2 astrocytomas (WHO 2016) (60).

#### 4.2.4 Outcome

There is huge variability in terms of survival among *IDH*-wt, morphologically grade 2, astrocytomas. A recently published meta-analysis examined data from 3,204 patients including 556 *IDH-*wt astrocytomas. In the entire cohort, the OS varied from a minimum of 87 to a maximum of 218 months (mean 118 months) for *IDH-*mut patients. On the contrary, *IDH*-wt astrocytomas showed shorter survival (from 9 to 120, mean 59 months). To date, the molecular profile resulted in a strong independent prognostic factor in the univariate analysis. EOR’s higher rate improved prognosis, while adjuvant therapies did not seem to impact the outcome in the *IDH*-wt population (39–43).

## 5 General Conclusions and Future Perspectives

Despite new evidence on the prognostic role of molecular features, there is no certainty about adequate therapeutic strategies, due to the lack of both perspective data, as well as technical limits of many oncological centers not performing an entire molecular analysis. According to the available studies, the *IDH-*mut subgroup achieves a demonstrable survival benefit by adjuvant chemoradiotherapy, as opposed to the *IDH*-wt counterpart (mOS 22 vs. 120 months) (13, 34, 42, 46, 49). Moreover, targeted therapy might also become a new potential treatment strategy. Indeed, encouraging results from basket trials showed clinically meaningful efficacy in gliomas harboring BRAF V600E mutation or NTRK 1/2/3 fusions, although missing enough data about grade 2 astrocytoma (73, 74). Currently, as previously mentioned, since these data derive from retrospective cohorts of patients treated according to clinicians’ choice, American Society of Clinical Oncology (ASCO)–SNO guidelines were found to be really helpful and affordable for the therapeutic management of diffuse astrocytic and oligodendroglial tumors in adults (50). In conclusion, while waiting for highly awaited new perspective trials, a multidisciplinary tumor board including an expert neuro-oncologist is strictly needed to plan a proper post-surgical therapeutic strategy for grade 2 astrocytomas, especially if *IDH*-wt.

## Author Contributions

VI conceived the analysis and wrote the paper. GT, PDS, and LS designed the figures and tables. MT and CP revised the paper and performed the English editing.

## Funding

This work was supported by the Apulia Region "Oncogenomic" Project.

## Conflict of Interest

The authors declare that the research was conducted in the absence of any commercial or financial relationships that could be construed as a potential conflict of interest.

## Publisher’s Note

All claims expressed in this article are solely those of the authors and do not necessarily represent those of their affiliated organizations, or those of the publisher, the editors and the reviewers. Any product that may be evaluated in this article, or claim that may be made by its manufacturer, is not guaranteed or endorsed by the publisher.
